# Diagnosis and treatment of the short-arm type posterior semicircular canal BPPV^☆^

**DOI:** 10.1016/j.bjorl.2020.10.012

**Published:** 2020-11-23

**Authors:** Lin Ping, Zhou Yi-fei, Wu Shu-zhi, Zheng Yan-yan, Yang Xiao-kai

**Affiliations:** a Wenzhou Third Clinical Institute Affiliated to Wenzhou Medical University, Third Affiliated Hospital of Shanghai University, Wenzhou People’s Hospital, Neurology Department, Wenzhou, Zhejiang, China; b Wenzhou Third Clinical Institute Affiliated to Wenzhou Medical University, Third Affiliated Hospital of Shanghai University, Wenzhou People’s Hospital, ENT Department, Wenzhou, Zhejiang, China

**Keywords:** Benign paroxysmal positional vertigo, Posterior, Semicircular canal, Lithiasis, Therapy

## Abstract

**Introduction:**

The Epley maneuver is useful for the otoconia to return from the long arm of the posterior semicircular canal into the utricle. To move otoconia out of the posterior semicircular canal short arm and into the utricle, we need different maneuvers.

**Objective:**

To diagnose the short-arm type BPPV of the posterior semicircular canal and treat them with bow-and-yaw maneuver.

**Methods:**

171 cases were diagnosed as BPPV of the posterior semicircular canal based on a positive Dix–Hallpike maneuver. We first attempted to treat patients with the bow-and-yaw maneuver and then performed the Dix–Hallpike maneuver again. If the repeated Dix–Hallpike maneuver gave negative results, we diagnosed the patient with the short-arm type of BPPV of the posterior semicircular canal and considered the patient to have been cured by the bow-and-yaw maneuver; otherwise, probably the long-arm type BPPV of the posterior semicircular canal existed and we treated the patient with the Epley maneuver.

**Results:**

Approximately 40% of the cases were cured by the bow-and-yaw maneuver, giving negative results on repeated Dix–Hallpike maneuvers, and were diagnosed with short-arm lithiasis.

**Conclusion:**

The short-arm type posterior semicircular canal BPPV can be diagnosed and treated in a convenient and comfortable manner.

## Introduction

**Figure 1:**
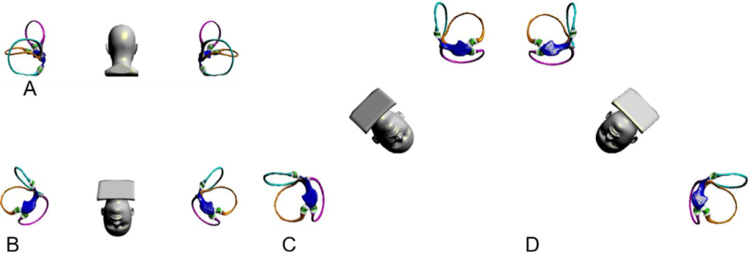
Bow-and-yaw maneuver. (A) Sit/kneel upright, posterior view. The membranous semicircular canals of the left and right ears, including cupula, are shown. (B) Holds and bows the patient’s head 135°. (C) Rotate the patient’s head 45° to the right. (D) Rotate the patient’s head 45° to the left. Shaking the head helps to shed the otolith before repeating C, D steps.

Benign paroxysmal positional vertigo (BPPV) is the most common cause of peripheral vertigo. BPPV is characterized by brief episodes of vertigo and nystagmus brought on by specific changes of head position relative to gravity and afflicts the posterior semicircular canal (PSC) specifically. The pathophysiology of this condition has been described by the theories of canalolithiasis (free-floating otoliths in the semicircular canals) and cupulolithiasis (otoconia are directly attached to the cupula).^1^, ^2^, ^3^ The vertical-torsional positional nystagmus evoked by the Dix–Hallpike maneuver indicates BPPV of the posterior or, rarely, the anterior semicircular canals, while horizontal positional nystagmus triggered by the supine roll maneuver is seen in BPPV of the horizontal semicircular canals.^4^ The PSC is the most frequently involved structure because of its anatomical position. Since otoconia are thought to be located in the long arm of the posterior canal, particle-repositioning maneuvers, such as the Epley maneuver, are useful to return the otoconia from the long arm of PSC into the utricle.^5^ However, Oas has proposed a subtype of PSC-BPPV, known as the short-arm type of BPPV, in which the otoconia are assumed to be located in the short arm (between the cupula and the utricle).^6^ Anatomically, the most vulnerable site is the short arm of the PSC because of its conical shape and its position directly below the utricle when the patient is upright.^7^ To move otoconia out of the short arm of the PSC, a substantially different process from moving them of the long arm, clinicians require different maneuvers.^8^, ^9^, ^10^ The present study aims to redesign the diagnosis and treatment strategy of PSC-BPPV, not only to determine which semicircular canal is affected but also to determine whether the otoconia are in the short arm or the long arm. It is convenient to treat short-arm lithiasis with the bow-and-yaw maneuver (Fig. 1) and long-arm lithiasis with the Epley maneuver.

## Methods

### Physical simulation

The three-dimensional membrane labyrinth model was established in standard space coordinate system,^11^ and the otolith in different positions of the membranous labyrinth was set up. The 3-dimensional physical simulation of this study uses the open-source bullet physical engine, the rendering engine uses the open-source cycles rendering engine based on physical reality restoration, and the development environment is the open-source 3D software Blender v2.79b. The physical engine needs to set variables such as friction and gravity. Physical parameter setting of semicircular canel: add selected objects as rigid body: passive, change collision shapes as object: mesh, set surface response friction to 0.5, bounce force to 0, sensitivity margin to 0.04.The scene gravity option uses the default value of −9.810 on the Z axis.

**Figure 2:**
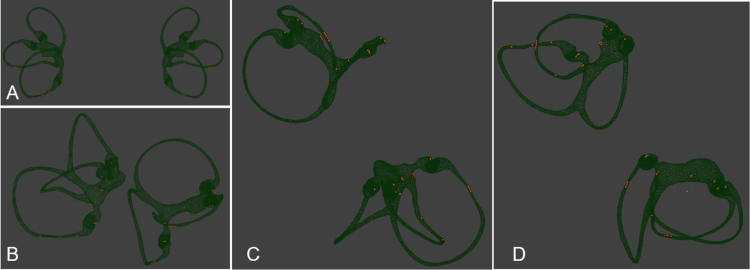
Otolith movement observation of Dix–Hallpike maneuver. (A) Sit upright, posterior view. The otoliths in different positions of the membranous labyrinth were set up. (B) Rotate the patient’s head 45° to the right. (C) Moving the patient to supine lying with the neck extended 30° (side view). (D) Moving the patient to supine lying with the neck extended 30° (posterior view).

We program with python language to rotate the labyrinth model to the specified position, and start the physical engine until the movement of otolith stops. The otolith movement in different positions during the Dix–Hallpike maneuver (Fig. 2) and Epley maneuver were analyzed based on the physical engine, and we observe whether the otolith on the short arm side and long arm side of the posterior semicircular canal can be reduced to the utricle.

**Figure 3:**
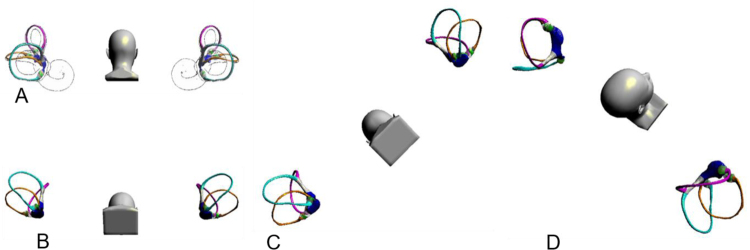
Supine Dix–Hallpike maneuver. (A), Sit upright, posterior view. The bony and membranous semicircular canals of the left and right ears, including cupula, are shown. (B) Bows the patient’s head 60°. (C) Rotate the patient’s head 45° to the right. (D) Moving the patient to supine lying without the neck extended.

**Figure 4:**
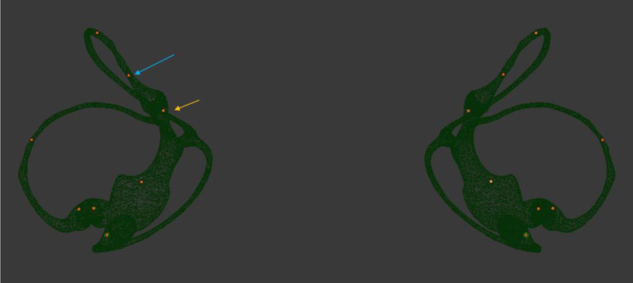
Otolith movement observation of Bow-and-yaw maneuver. Bows head 135°. The blue arrow refers to the otolith on the long arm side of PSC, the orange arrow refers to the otolith on the short arm side of PSC. Based on the physical engine, the otolith on the short arm side can be reduced to the utricle directly through the short arm and the otolith on the long arm side cannot be reduced to the utricle.

According to the theory of otolith, the Dix–Hallpike maneuver was modified to make it easy to operate (Fig. 3), the bow-and-yaw maneuver (Fig. 1) was designed specifically targeted to return the otolith in short-arm side back to utricle. The otolith movement during the bow-and-yaw maneuver was analyzed based on the physical engine, and the test is repeated 10 times to confirm that the otolith on the short arm side can be reduced to the utricle and the otolith on the long arm side cannot be reduced to the utricle (Fig. 4).

### Clinical research

A self-controlled study with consecutive PSC-BPPV patients who visited the vertigo clinic at Wenzhou People’s Hospital, China, during the period from August 2017 to December 2019.

171 individuals included in the study, including 57 males and 114 females aged 53 ± 14 years, between 24 and 83. The time since onset was 1 h to 6 years (median 2-weeks). The main purpose of the study is to determine whether the bow-and-yaw maneuver can treat the short arm type posterior semicircular canal BPPV, as the Epley maneuver can treat the long arm type posterior semicircular canal BPPV.

### Inclusion criteria

1) Positional vertigo or dizziness repeatedly occurs when the patient is lying down.
2) Dix–Hallpike maneuver induces torsional nystagmus, and supine roll maneuver is negative or induces torsional nystagmus.
3) Not attributable to other illnesses.

### Exclusion criteria

1) Age >83 years;
2) Physical therapy cannot be completed due to poor language understanding or compliance;
3) Patients with serious cervical spondylosis, arrhythmia, heart failure, dyskinesia, upper gastrointestinal bleeding.

### Diagnosis and treatment strategy

Initially, PSC-BPPV was diagnosed as usual and then treated with the bow-and-yaw maneuver. The diagnosis was short-arm lithiasis if the Dix–Hallpike maneuver became negative; otherwise, the condition may be long-arm lithiasis and should be treated with the Epley maneuver, or some cases of failed bow-and-yaw maneuver still represent short-arm disease.

### Step 1: diagnosis of PSC-BPPV

We inquired about the patient’s medical history, conducted routine physical and neurological examinations, and performed the supine Dix–Hallpike maneuver and supine roll maneuver.

PSC-BPPV was considered when the supine Dix–Hallpike maneuver induced upbeat torsional nystagmus and the supine roll maneuver gave a negative result or induced torsional nystagmus. If nystagmus is atypical, the Dix–Hallpike maneuver should be repeated.

### Step 2: treatment for short-arm type PSC-BPPV

After the diagnosis of PSC-BPPV is established, there is still no direct way to distinguish short-arm lithiasis from long-arm lithiasis. We used diagnostic therapy for differential diagnosis. We first attempted to treat patients with the bow-and-yaw maneuver, which works only for short-arm lithiasis. Subsequently, the Dix–Hallpike maneuver was performed again. We deduced that otoconia were present in the short arm if the Dix–Hallpike maneuver gave a negative result; otherwise, we deduced the presence of otoconia in the long arm, and rarely in the short arm side failed to respond to bow-and-yaw maneuver.

### Step 3: treatment for long-arm type PSC-BPPV

To cure long-arm type PSC-BPPV, we performed the Epley maneuver, which we repeated until the Dix–Hallpike maneuver gave a negative result.

### Statistical analysis

The data generated in the study were analyzed in R (R Core Team, 2020). McNemar’s test was used to evaluate bow-and-yaw maneuver’s therapeutic effect.

### Standard protocol approvals, registrations, and patient consents

The study was approved by the Ethics Committee of Wenzhou People’s Hospital (nº 2018100) and informed consent was obtained from all patients.

### Data availability statement

Physical simulation result recoded as gif and the three-dimensional membrane labyrinth model are available within the supplementary materials.

## Result

### Improvement of diagnostic maneuver

The traditional Dix–Hallpike maneuver needs to adjust the sitting position, tilt the head back below the table, and the operation is not convenient; more importantly, the bottom of the posterior semicircular canal is flat and the otoliths are scattered which will lead to the intensity of nystagmus induced by Dix Hallpike maneuver, which varies and affects the diagnostic sensitivity (Fig. 2).

The Dix Hallpike maneuver was modified and named Supine Dix–Hallpike maneuver; the head was first bent forward for 60°, then turned back for 45° on one side, then laid down, and the head was not tilted back (Fig. 3). Its advantages are: (1) the head was bent forward for 60°, the otolith in long arm side of the PSC slid to the place near the ampulla, so the otolith motion distance induced by the diagnosis maneuver was longer, the induced nystagmus was more obvious, and the diagnostic sensitivity was theoretically higher. (2) The head was not tilted back, and the operation was simple and easy. (3) The otolith on the long arm side of the anterior semicircular canal is more difficult to induce to leave the ampullar region, and the identification is easier. (4) Reduction of otolith movement in the contralateral posterior semicircular canal and reduced a positive result of healthy side Dix Hallpike maneuver.

The Dix–Hallpike maneuver induced upbeat rotational nystagmus in all 171 patients, six of which was decisive on both sides. Rotational nystagmus was induced by ipsilateral supine roll maneuver in 60 cases and contralateral supine roll maneuver in 2 cases, and the degree of vertigo was significantly less than that induced by the Dix–Hallpike maneuver.

**Figure 5:**
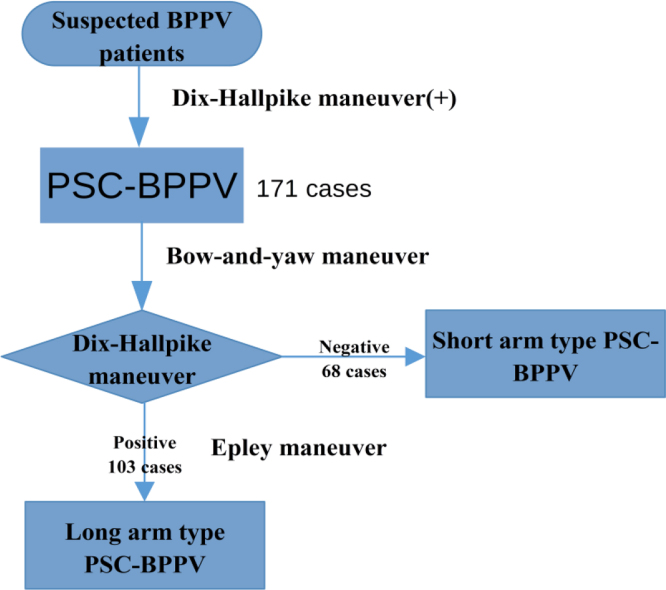
Diagnosis and treatment strategy for the short-arm type PSC-BPPV. Try reduction otolith in the short arm of the PSC with bow-and-yaw maneuver, if failed, reduction otolith in the long arm of the PSC with Epley maneuver.

When we performed the Dix–Hallpike maneuver again after the bow-and-yaw maneuver, 68 cases gave negative results, accounting for 40% of the total, and those cases were considered to be short-arm PSC-BPPV; the other 103 cases gave positive results, including three cases in which nystagmus decreased significantly (Fig. 5). The cases with positive results were treated with the Epley maneuver, which succeeded after a single application in 76 cases and after 2–5 applications in the other cases, except for one case of treatment failure. There was virtually no significant discomfort during the bow-and-yaw maneuver, except for few patients with mild dizziness. One patient with left-sided PSC-BPPV converted to right side PSC-BPPV after the bow-and-yaw maneuver and was cured by the right Epley maneuver. The effective rate of nearly 40% is enough to explain that the bow-and-yaw maneuver has a therapeutic effect on BPPV patients.

## Discussion

The pathogenesis of BPPV is still not well understood. In 1921, Barany first described a case of BPPV and incorrectly attributed the condition to an otolithic disturbance, because the dizziness was precipitated by head position. In 1952, Dix and Hallpike conducted a systematic study of this disorder and proposed the Dix–Hallpike maneuver for the diagnosis of BPPV, which has been used up to now. Dix and Hallpike proposed the concept of BPPV but incorrectly concluded that BPPV results from an otolithic disturbance.^12^ Cupulolithiasis was described in 1969 by Schuknecht^1^ as a condition in which degenerative debris in the endolymph is deposited on the short arm side of the PSC and adheres to the utricular side of the cupula. In contrast, canalithiasis was described in 1979 by Hall,^2^ who reported that free-floating dense particulate matter in the long arm of the PSC causes BPPV. The two lithiasis theories are complementary to each other, and BPPV was finally attributed to semicircular canal lesions. Epley’s canalith repositioning maneuver, which is designed according to the theory of canalithiasis, uses gravity to guide the debris out of the long arm of the PSC into the utricle. The Epley maneuver is an excellent treatment option that can cure most BPPV, making the canalithiasis theory widely accepted.^3^ In fact, the initial lithiasis theory was limited to the PSC. Recently, other types of semicircular canal BPPV have gradually been recognized, and each is divided into cupulolithiasis and canalithiasis. Because the short arm of the PSC is positioned directly below the utricle when the patient is upright, the detached otoliths should easily settle under the effect of gravity, leading to short arm lithiasis.^7^ Therefore, the otolith can be in the long arm or the short arm of the semicircular canal and can also be attached to the cupula. For horizontal semicircular canal BPPV, according to the traditional concept, we can distinguish between canalithiasis and cupulolithiasis based on the nystagmus induced by the supine roll maneuver, specifically, whether it beats toward the lower ear (geotropic) or the upper ear (apogeotropic). According to the lithiasis theory, the former nystagmus indicates otoconia in the long arm, while the latter nystagmus indicates otoconia in ampullar region, whether attached to the cupula or float in the endolymph and can be on the the short arm side or long arm side. For the PSC-BPPV, there have been few reported cases of cupulolithiasis, which is inconsistent with the lithiasis theory.

The vertical-torsional nystagmus evoked by the Dix–Hallpike maneuver indicates BPPV of the posterior or, rarely, the anterior semicircular canals but is difficult to distinguish by upward vertical/torsional nystagmus and downward vertical/torsional nystagmus. The supine Dix–Hallpike maneuver, with the head straight while lying down leads to otolith floating only in the PSCs and not in the anterior semicircular canals. Therefore, the vertical-torsional nystagmus evoked by the supine Dix–Hallpike maneuver specifically indicates PSC-BPPV.

It is essential to distinguish short-arm type BPPV and long-arm type BPPV because the path otoliths return to utricle is completely different and requires different therapy maneuvers.^8^, ^9^, ^10^ In contrast, whether the otolith is floating in the semicircular canal or adhering to the cupula, the choice of therapy maneuver is nearly the same. However, there is currently a lack of systematic and in-depth research on how to distinguish between short-arm type BPPV and long-arm type BPPV.

Since 2001, there has been a recognized need to specify where the otoconia exist within the affected semicircular canal system and differentiate short-arm from long-arm canalolithiasis.^6^ Data based on videonystagmography recordings suggest that two pathological conditions can often be differentiated by the characteristics of the positioning nystagmus observed in the head-hanging position after the paroxysmal part of the nystagmus has ended. Short-arm canalolithiasis has persistent unidirectional nystagmus, while long-arm canalolithiasis has a small transient contra-directional nystagmus.^6^ In 2011, Taura reported four cases of short-arm type PSC-BPPV and noted that the reversal of nystagmus was not or very faintly observed during sitting up from the Dix–Hallpike position.^9^ Buki reported that the unilateral sitting up vertigo/body sway felt/shown by the patients during sitting up from the Dix–Hallpike position is more common in short-arm canalolithiasis than in long-arm canalolithiasis.^10^, ^13^ For therapy, repetitive sit-ups from the Dix–Hallpike positions,^10^ application of vibration to the mastoid process of the affected ear with the healthy ear positioned downwards,^9^ and positioning the head upside down^8^ are recommended to return the otoconia from the short arm into the utricle.

According to the classification formulated by the Committee for Classification of Vestibular Disorders of the Bárány Society in 2017, limited to the current knowledge of clinical aspects and pathomechanisms of BPPV, loose otoconia within the short arm of the semicircular canals (on the utricular side of the cupula) need to be addressed in the future.^14^

In 2012, Foster designed a novel self-administered exercise, the half somersault: first tilt the head up and back, and then place the head in somersault position. The subjects reported more dizziness during the Epley than during the half somersault exercise.^15^ Although Foster believes that the reason for the effectiveness of this maneuver is to reduce the otolith on the long arm side, it is more likely to reduce the otolith on the short arm side.

Unlike previous reports, our study shows that short-arm type PSC-BPPV, which is not uncommon, makes up approximately half of the total. There is no doubt about the way to diagnose PSC-BPPV, but only about the way to distinguish short-arm type BPPV from long-arm type BPPV. As shown in Fig. 1, the bow-and-yaw maneuver can reposition otoconia from the short arm of the PSC to the utricle.

To make sure the otolith on the long arm side cannot be reduced to the utricle by bow-and-yaw maneuver, repeat the test for 10 times based on the physical engine.

The otolith movement during the bow-and-yaw maneuver was analyzed based on the physical engine and repeated for 10 times; the results show that the otolith on the long arm side cannot be reduced to the utricle. This gives us sufficient reason to make the following judgment: once PSC-BPPV has been confirmed, if the Dix–Hallpike maneuver gives a negative result just following the completion of the bow-and-yaw maneuver, otoconia in the short arm should be considered; otherwise, otoconia in the long arm should be considered, but occasionally, there may be otoconia in the short arm that have failed to return to the utricle during the bow-and-yaw maneuver.

The Epley maneuver is an effective treatment for PSC-BPPV. The sequential head movements of the Epley maneuver cause otoconial debris to move from the long arm to the utricle. As the cure rate of the Epley maneuver for PSC-BPPV is significantly higher than the ratio of the long-arm type PSC-BPPV, it is doubtful whether the Epley maneuver works for short-arm type PSC-BPPV. It is true that when the head is turned to the face-down position, otoconial debris may move from the short arm to the utricle. Even so, the Epley maneuver is not an excellent choice for short-arm type PSC-BPPV, and it will inevitably cause dizziness and vertigo. Remarkably, the bow-and-yaw maneuver can cure the short-arm type of PSC-BPPV without significant discomfort. More importantly, the procedure is easy to perform and does not even require identification of the affected side; thus, this method is suitable for self-treatment of PSC-BPPV.

## Conclusion

Although this study used only a small sample of cases and there is a need for large-scale clinical validation, our findings are sufficient to demonstrate that short arm lithiasis can be diagnosed and treated in a convenient and comfortable manner.

## Funding

Study funded by 10.13039/501100007194Wenzhou Municipal Science and Technology Bureau (Grant nº ZS2017020, Y20180626), and 10.13039/501100004731Natural Science Foundation of Zhejiang Province (Grant nº LSY19H090002).

## Conflict of interest

The authors declare no conflicts of interest
